# Association of OX40L Polymorphisms with Sporadic Breast Cancer in Northeast Chinese Han Population

**DOI:** 10.1371/journal.pone.0041277

**Published:** 2012-08-03

**Authors:** Yuan Weiguang, Li Dalin, Xu Lidan, Cai Yonggang, Chen Shuang, Liu Yanhong, Xu Fengyan, Fu Zhenkun, Pang Da, Li Dianjun

**Affiliations:** 1 Department of Immunology, Harbin Medical University, Harbin, China; 2 Department of Tumor Cell Biology, Institute of Cancer Prevention and Treatment, Harbin Medical University, Harbin, China; 3 Department of Surgery, The Third Affiliated Hospital of Harbin Medical University, Harbin, China; 4 Laboratory of Medical Genetics, Harbin Medical University, Harbin, China; 5 Department of Laboratory Medicine, The Second Hospital of Harbin Medical University, Harbin, China; Sudbury Regional Hospital, Canada

## Abstract

OX40L is an important costimulatory molecule that plays a crucial role in the regulation of T-cell-mediated immunity. The interaction of OX40-OX40L is involved in the pathogenesis of multiple autoimmune and inflammatory diseases such as systemic lupus erythematosus (SLE), carotid artery disease and cancer. The genetic variants of OX40L can increase the risk of SLE, atherosclerosis, systemic sclerosis and show gender-specific effects in some studies. Accordingly, we performed a case-control study including 557 breast cancer patients and 580 age- and sex-matched healthy controls to investigate whether single nucleotide polymorphisms (SNPs) in the OX40L gene are associated with sporadic breast cancer susceptibility and progression in Chinese Han women. Seven SNPs of OX40L (rs6661173, rs1234313, rs3850641, rs1234315, rs12039904, rs844648 and rs10912580) were genotyped with the method of polymerase chain reaction-restriction fragment length polymorphism (PCR-RFLP). The results indicated that rs3850641G allele could increase the susceptibility to breast cancer (*P* = 0.009662), even in the validation study (*P* = 0.0001515). A significant association between rs3850641 and breast cancer risk was observed under the additive model and dominant model (*P* = 0.01042 and 0.01942, respectively). The haplotype analysis showed that haplotype A_rs844648_A_rs10912580_ was significantly associated with breast cancer, even after 10,000 permutations for haplotypes in block only (*P* = 0.0003). In clinicopathologic features analysis, the association between rs1234315 and C-erbB2 status was significant (*P* = 0.02541). Our data primarily indicates that rs3850641 of OX40L gene contributes to sporadic breast carcinogenesis in a northeast Chinese Han population.

## Introduction

OX40L (known as TNFSF4, CD252), the cognate ligand of OX40, is a member of the tumor necrosis factor superfamily. The OX40L gene is located on human chromosome 1 and encodes a type II glycoprotein which expressed not only on professional antigen-presenting cells (APCs), but also on CD4+T cells, CD8+T cells, vascular endothelial cells, mast cells and activated NK cells [1], [2], [3], [4], [5]. The interaction between OX40 and OX40L provides a costimulatory signal that strongly regulates the proliferation and survival of T lymphocytes, modulates NKT cell and NK cell function, and contributes to the differentiation and activity of regulatory T cells [6], [7]. Antitumor immunity provides a protective barrier to tumor formation and progression [8]. Accumulating evidence indicates that inflammatory response plays a decisive role at different stages of tumor development and contributes to the initiation and progression of cancer. Moreover, avoiding immune destruction was considered as an emerging hallmark of cancer [9], [10].

Breast cancer is the most common invasive malignancy. It is estimated that more than one million women are diagnosed with breast cancer and over 450,000 dead worldwide every year [11]. In the past decades, several studies have suggested that OX40L involved in the initiation and progression of breast cancer. OX40L fusion protein significantly inhibited the growth of mouse 4T1 breast tumor model [12] and breast cancer cell-derived thymic stromal lymphopoietin (TSLP) contributes to breast tumor development by inducing OX40L expression on DCs [13]. However, there is no report about the association of OX40L gene variation with the risk for breast cancer.

Single nucleotide polymorphisms (SNPs) association analysis reveals the predisposition of diverse diseases in different ethnic groups and benefits to develop effective risk assessment and treatment strategies. So far, previous studies were mainly focused on the association of OX40L polymorphisms with autoimmune diseases and inflammatory diseases such as systemic lupus erythematosus (SLE), systemic sclerosis (SSc) and myocardial infarction (MI) [14], [15], [16], [17], [18], [19]. Interestingly, some results demonstrated gender-specific effects in the associations of OX40L variants with atherosclerosis, MI in females than in males [16], [20]. Therefore, in this study, we sought to determine if the polymorphisms of OX40L gene are associated with sporadic breast cancer in northeast Chinese Han population.

## Results

### Frequencies of Alleles and Genotypes between Cases and Controls

The clinical features of cases with breast cancer are summarized in Table 1. The genotype distribution of the seven SNPs was in HWE in controls (*P*>0.05). About 5% samples were randomly selected for direct DNA sequencing, and the reproduction rate was 100%. As shown in Table 2, a higher prevalence of rs3850641G allele was observed in breast cancer patients (14.27%) than in controls (10.69%), which showed a statistically significant association between rs3850641G allele and breast cancer risk (*P* = 0.009662, OR = 1.391, 95%CI = 1.083–1.787) calculated by the Chi-square test with Plink 1.07 software. In the validation study, the association remained significant between rs3850641G allele and breast cancer risk (*P* = 0.0001515, OR = 1.63, 95%CI = 1.264–2.102), even after correction with 10,000 permutations for single markers only (*P* = 0.0003). The rs844648A allele was associated with an increased risk of breast cancer in the study cohort (*P* = 0.03616, OR = 1.193, 95%CI = 1.011–1.408). However, we failed to replicate this result in the validation cohort (*P* = 0.1997, OR = 1.123, 95%CI = 0.9404–1.342). No significant differences were found between the other SNPs and breast cancer risk.

**Table 1:** Clinicalpathologic features of breast cancer cases.

Clinicalpathologic features	Case n (%)
Tumor type	
IDC	451 (80.97)
ILC	13 (2.33)
Intraductal carcinoma	41 (7.36)
Mucinous adenocarcinoma	9 (1.62)
Others	43 (7.72)
Tumor size	
with the diameter less than 2 cm	185 (33.21)
with the diameter of 2 to 5 cm	243 (43.63)
with the diameter more than 5 cm	26 (4.67)
Unknown	103 (18.49)
LN involvement	
Positive	232 (41.65)
Negative	307 (55.12)
Unknown	18 (3.23)
ER	
Positive	277 (49.73)
Negative	199 (35.73)
Unknown	81 (14.54)
PR	
Positive	342 (61.40)
Negative	132 (23.70)
Unknown	83 (14.90)
P53	
Positive	145 (26.03)
Negative	313 (56.19)
Unknown	99 (17.78)
C-erbB-2	
Positive	185 (33.21)
Negative	286 (51.35)
Unknown	86 (15.44)

Abbreviations: IDC = infiltrative ductal carcinoma; ILC = infiltrative lobular carcinoma; LN = lymph node; TZ = tumor size; ER = estrogen receptor; PR = progesterone receptor; P53 = tumor protein 53; C-erbB2 = human epidermal growth factor receptor 2.

**Table 2:** Alleles of the seven SNPs of OX40L gene between cases and controls.

SNP	Minor allele	Study cohort				Validation cohort			
		Cases n = 557	Controlsn = 580	*P* value	OR(95%CI)	Cases n = 507	Controlsn = 492	*P* value	OR(95%CI)
rs6661173	A	0.06463	0.05776	0.494	1.127(0.7996–1.589)				
rs1234313	G	0.3402	0.3466	0.7504	0.9723(0.8177–1.156)				
rs3850641	G	0.1427	0.1069	**0.009662**	**1.391(1.083–1.787)**	0.1746	0.1148	**0.0001515**	**1.63(1.264–2.102)**
rs1234315	T	0.5144	0.4802	0.1031	1.147(0.9727–1.352)				
rs12039904	T	0.2756	0.2948	0.3097	0.9099(0.7583–1.092)				
rs844648	A	0.4722	0.4284	**0.03616**	**1.193(1.011–1.408)**	0.4369	0.4085	0.1997	1.123(0.9404–1.342)
rs10912580	G	0.281	0.2914	0.583	0.9503(0.7922–1.14)				

SNP, single nucleotide polymorphism. Allele data and basic allelic Chi-square test were analyzed using Plink 1.07. Asymptotic *P* value, estimated odds ratio (OR) and 95% confidence interval (CI) were calculated. Significant values (*P*<0.05) are in bold.

The association analysis between genetic models and breast cancer risk demonstrated a moderate association between rs3850641 and breast cancer risk under the additive model (*P* = 0.01042) and dominant model (*P* = 0.01942) using a logistic regression analysis with Plink 1.07 software. The association was replicated in the validation study under the additive model (*P* = 0.0001348) and dominant model (*P* = 0.000059) (Table 3). Although a moderate association was demonstrated between rs844648 and breast cancer risk under the additive model (*P* = 0.03836) and dominant model (*P* = 0.03459) in the study cohort, this result failed to be confirmed in the validation cohort (Table 3).

**Table 3:** Genotyping and genetic models of OX40L gene SNPs in cases and controls.

SNP	Genetic Models	Study cohort				Validation cohort			
		Cases^a^	Controls^b^	*P* value	OR (95%CI)	Cases^c^	Controls^d^	*P* value	OR (95%CI)
rs6661173	additive	1/70/486	4/59/517	0.4967	1.126(0.8–1.584)				
	dominant	71/486	63/517	0.3249	1.199(0.8355–1.72)				
	recessive	1/556	4/576	0.2276	0.259(0.02886–2.324)				
rs1234313	additive	62/255/240	68/266/246	0.7485	0.9718(0.8162–1.157)				
	dominant	317/240	334/246	0.8183	0.9728(0.7691–1.231)				
	recessive	62/495	68/512	0.7534	0.9431(0.6542–1.359)				
rs3850641	additive	13/133/411	6/112/462	**0.01042**	**1.387(1.08–1.782)**	10/157/340	7/99/386	**0.0001348**	**1.662(1.281–2.158)**
	dominant	146/411	118/462	**0.01942**	**1.391(1.055–1.834)**	167/340	106/386	**0.000059**	**1.789(1.347–2.375)**
	recessive	13/544	6/574	0.09628	2.286(0.8628–6.057)	10/497	7/485	0.5038	1.394(0.5264–3.692)
rs1234315	additive	153/267/137	132/293/155	0.1059	1.144(0.9718–1.348)				
	dominant	420/137	425/155	0.4117	1.118(0.8565–1.459)				
	recessive	153/404	132/448	0.06728	1.285(0.9823–1.682)				
rs12039904	additive	36/235/286	54/234/292	0.3072	0.9089(0.7566–1.092)				
	dominant	271/286	288/292	0.7356	0.9607(0.7613–1.212)				
	recessive	36/521	54/526	0.07697	0.6731(0.434–1.044)				
rs844648	additive	124/278/155	112/273/195	**0.03836**	**1.189(1.009–1.402)**	99/245/163	92/218/182	0.2111	1.117(0.9391–1.329)
	dominant	402/155	385/195	**0.03459**	**1.314(1.02–1.692)**	344/163	310/182	0.1078	1.239(0.9542–1.609)
	recessive	124/433	112/468	0.2202	1.197(0.8981–1.594)	99/408	92/400	0.7395	1.055(0.7695–1.446)
rs10912580	additive	40/233/284	53/232/295	0.5829	0.9503(0.7921–1.14)				
	dominant	273/284	285/295	0.9663	0.995(0.7885–1.256)				
	recessive	40/517	53/527	0.2298	0.7693(0.5014–1.18)				

SNP, single nucleotide polymorphism.

a the number of cases in study cohort was 557, ^b^the number of controls in study cohort was 580, ^c^the number of cases in validation cohort was 507, and, ^d^the number of controls in validation cohort was 492.

The *P* value, odds ratio (OR) and 95% confidence interval (CI) in each comparison were assessed under an additive model (additive effect of having one additional copy of a allele, a was for the minor allele and A was for the major allele), dominant model (aa+Aa vs. AA), and recessive model (aa vs. Aa+AA) using logistic regression adjusted for age with Plink 1.07. Significant values (*P*<0.05) are in bold.

### Frequencies of Haplotypes between Cases and Controls

We further analyzed the association between haplotypes and breast cancer risk. With the method described in the statistical analysis section, three blocks were identified using the Haploview program 4.1 based on the Solid Spine of LD method. As shown in Fig. S1, rs6661173 and rs1234313 (D’ = 0.87) belonged to the LD block 1, and they constructed three haplotypes (G_rs6661173_A_rs1234313_, G_rs6661173_G_rs1234313_ and A_rs6661173_G_rs1234313_), which were not associated with breast cancer risk (*P*>0.05, Table 4). Rs3850641, rs1234315 and rs12039904 (D’ ≥0.83) constructed the LD block 2 containing five haplotypes. The haplotype A_rs3850641_C_rs1234315_C_rs12039904_ was the most common haplotype (45.9% in cases and 49.4% in controls), and haplotype A_rs3850641_T_rs1234315_T_rs12039904_ was less frequent appeared (25.1% in cases and 27.0% in controls). The haplotype G_rs3850641_T_rs1234315_C_rs12039904_ had a higher frequency in cases (13.4%) than in controls (10.4%) and the difference was significant (*P* = 0.0285). Rs844648 and rs10912580 (D’ = 0.91) involved in the LD block 3 constructed four haplotypes. The haplotype A_rs844648_A_rs10912580_ had a higher frequency in cases (21.1%) than in controls (14.6%) and the difference was significant (*P* = 0.000049773), even after correction with 10,000 permutations for haplotypes in blocks only (*P* = 0.0003). The association between breast cancer risk and haplotype G_rs844648_A_rs10912580_ as well as haplotype G_rs844648_G_rs10912580_ was significant (*P* = 0.009, *P* = 0.0267, respectively).

**Table 4:** Haplotype of OX40L gene SNPs in cases and controls.

Block	Haplotype	Freq.	Case,Control Frequencies	Chi-square	*P* value	*P*_permutation_ *
Block 1 (rs6661173 -rs1234313)	GA	0.652	0.656, 0.647	0.22	0.6392	0.9998
	GG	0.287	0.279, 0.295	0.732	0.3921	0.987
	AG	0.056	0.061, 0.051	1.05	0.3055	0.9595
Block 2 (rs3850641 –rs1234315 -rs12039904)	ACC	0.477	0.459, 0.494	2.78	0.0955	0.5543
	ATT	0.26	0.251, 0.270	1.084	0.2979	0.9532
	GTC	0.119	0.134, 0.104	4.797	**0.0285**	0.1943
	ATC	0.115	0.126, 0.105	2.449	0.1176	0.6282
	ACT	0.023	0.021, 0.024	0.187	0.6651	0.9999
Block 3 (rs844648 -rs10912580)	GA	0.536	0.508, 0.563	6.815	**0.009**	0.0651
	AG	0.272	0.262, 0.283	1.301	0.2541	0.9196
	AA	0.177	0.211, 0.146	16.457	**0.000049773**	**0.0003**
	GG	0.014	0.019, 0.009	4.913	**0.0267**	0.1841

Blocks were constructed according to the value of D’ generating from our own data and Haplotype was defined as the method of solid spine of LD. Haplotype data was analyzed using Haploview 4.1.

* For accurate multiple testing correction, *P* values for haplotypes in blocks only were permutated 10,000 times by Haploview 4.1 program. Significant values (*P*<0.05) are in bold.

### Clinicopathologic Features

As shown in Table 1, the clinicopathologic features of 557 breast cancer patients are summarized, including histological grade, tumor size, lymph node metastasis and the status of estrogen receptor (ER), progesterone receptor (PR), epidermal growth factor receptor 2 (C-erbB2) and tumor protein 53 (P53) which were investigated in this study. As shown in Table S1, significant association was observed between rs1234315 and C-erbB2 status (*P*_allelic_ = 0.02541, *P*_dominant_ = 0.008199, *P*_additive_ = 0.02896). Moderate association was observed between rs3850641 and tumor size (*P*_allelic_ = 0.04853) as well as between rs844648 and histological grade (*P*_global_ = 0.022, data not shown). However, no statistical association was found in other clinicopathologic features (lymph node metastasis and the status of ER, PR and P53).

We further analyzed the association between haplotypes and clinicopathologic features. The frequency of A_rs3850641_C_rs1234315_C_rs12039904_ haplotype in the LD block 2 was higher in C-erbB2 positive cases (*P* = 0.0245) and G_rs844648_G_rs10912580_ haplotype in the LD block 3 had a lower frequency in C-erbB2 positive cases (*P* = 0.0362, Table S2). No significant association was observed in other clinicopathologic features.

## Discussion

Breast cancer is a highly heterogeneous malignancy with complex genetic patterns. Further understanding of the patient’s genetic background is critical for improving the ability to assess breast cancer and optimizing the approaches for prevention and treatment.

OX40L is an important member of the tumor necrosis factor superfamily. Costimulatory molecules play crucial roles in inflammation and antitumor immune responses. The Cross-linking of OX40L with OX40 provides a T-cell costimulatory signal, resulting in increased proliferation and cytokine production [3], [21]. Recent study suggested that OX40L could regulate the balance of Th cell polarization in different cytokine microenvironments [22]. And their antitumor effects are quite variable depending on the tumor microenvironments [13], [23]. Previous studies have shown that OX40L gene polymorphisms are associated with diverse autoimmune diseases. However, the association between SNPs of OX40L and breast cancer risk remain unknown. Therefore, we investigated the associations of seven OX40L SNPs with breast cancer risk. Our primarily data first demonstrate rs3850641G allele is associated with an increased risk of sporadic breast cancer in northeast Chinese women.

As known, rs3850641 is positioned in intron 1 of OX40L gene and found to be associated with MI [24], stroke in diabetic patients [25] and cardiovascular disease [26]. Furthermore, many studies indicated the associations were significant only in female patients [16], [24], [26]. It is notable that women generally exhibit more robust immunity than men and experience a generally greater risk for autoimmunity [27]. Recent reports indicated that co-stimulator B7-H1 seems to desensitize Tregs to estrogen-mediated functional reduction and play an important role in sex-related tumor immunity [28]. As a costimulatory molecule, OX40L also regulates the development and function of Tregs and decreases the number of tumor-infiltrating Tregs [29], [30], [31]. Consistent with these results, our study indicated that the rs3850641G allele can increase the susceptibility to breast cancer, and the additive and dominant model of rs3850641 showed association with breast cancer risk. A recent report by Smirnov and colleagues confirmed that there is a highly conserved binding site of miR-125b in the 3¢ end of OX40L. Ataxia telangiectasia mutated (ATM) gene regulates OX40L expression through miR-125b implicated in breast cancer and heart disease [32]. However, in the functional study, rs3850641 did not influence the binding of nuclear protein and the OX40L expression [24], which indicated that other molecular mechanisms may be involved in the SNP functions. Taken together, rs3850641 maybe contribute to the regulation of hormone-related pathways and play an important role in gender-specific disease, such as breast cancer.

Previous studies showed that rs844648 and rs12039904 were implicated in susceptibility to SSc [15] and SLE [18]. These two SNPs are located in the promoter region of OX40L gene where might effect the transcriptional efficiency. In our study, we found that the rs844648A allele was associated with an increased risk of breast cancer, and the association was significant under the additive and dominant models. However, this genetic association was not confirmed in our validation cohort. So, confirming this result with enlarged sample size was needed.

Recent study in GWAS demonstrated that different autoimmune diseases shared limited genetic overlap [33]. According to our results, we found that the direction of the observed associations were in agreement with previous findings in studies of cardiovascular disease, not in autoimmune diseases such as SLE. It suggested that the pathogenesis may be similar between of cardiovascular disease and breast cancer involved in the regulation of hormone-related pathways [34], which need to be estimated in further study.

In haplotype analysis, we found that haplotype G_rs3850641_T_rs1234315_C_rs12039904_ containing rs3850641G was associated with an increased risk of breast cancer. And haplotypes A_rs844648_A_rs10912580_ and G_rs844648_G_rs10912580_ may be risk factors in breast cancer, whereas haplotype G_rs844648_A_rs10912580_ may play a protective role in breast cancer. These results also suggested that rs3850641 may play an important role in the pathogenesis of breast cancer.

The clinicopathologic features analysis indicated that there are significant associations between rs1234315 and C-erbB2 status, as well as between the haplotypes A_rs3850641_C_rs1234315_C_rs12039904_ and G_rs844648_G_rs10912580_ and C-erbB2 status. C-erbB2 is an important factor in predicting the long-term survival of breast cancer patients. Overexpression of C-erbB2 leads to the activation of downstream signaling pathway associated with cell proliferation, differentiation and angiogenesis [35], which is associated with increased disease recurrence and worse prognosis [36]. This result may be correlated with the report that accumulating of Tregs at the tumor site is associated with poor prognosis [37], [38]. Altogether, rs1234315 may be important for the prognosis or prediction of breast cancer.

### Conclusion

Our primary data first demonstrates the genetic association of polymorphisms in OX40L gene with sporadic breast cancer. It suggests that rs3850641G is associated with an increased risk of breast cancer. OX40L gene polymorphisms may affect breast cancer risk and prognosis in Chinese Han population, northeast of China. It is necessary to confirm this result with enlarged sample size in multiethnic groups in the future.

## Materials and Methods

### Ethics Statement

The study is in compliance with the Helsinki declaration, and has been approved by the Medical Ethical Committee of Harbin Medical University. The informed written consent was obtained from all subjects.

### Subjects

In this study, we analyzed a total of 557 female sporadic breast cancer cases and 580 age- and sex-matched healthy controls. All subjects were recruited from the Department of Breast Surgery in the Third Affiliated Hospital of Harbin Medical University. All patients (mean age at 49.1±9.9 years) were pathologically confirmed, which pathological and clinical information were obtained from medical files (Table 1). The controls were frequency-matched to cases by age (mean age at 48.3±10.1 years) and without any history of personal or familial malignancy or autoimmune disorders. The validation cohort consisted 507 breast cancer cases (mean age at 49.4±10.1 years) and 492 age- and sex-matched healthy controls (mean age at 48.7±10.4 years), and the selection criteria for cases and controls was as described above. Both breast cancer cases and healthy controls were hereditarily unrelated and were recruited from Heilongjiang Province, northeast of China.

### SNP Selection and Genotyping

To determine the association between SNPs of OX40L and breast cancer risk, we selected SNP loci based on the published reports, in which these SNPs were significantly associated with autoimmune diseases and heart diseases or showed gender-specific effects in some studies. Then, we selected common and potentially functional SNPs with minor allele frequency (MAF) >0.10 located in the OX40L genes using NCBI dbSNP database. Finally, we discarded the SNPs which were in high linkage disequilibrium (LD) with each other in CHB population using HapMap database. Seven SNPs (rs6661173, rs1234313, rs3850641, rs1234315, and rs12039904, rs844648, rs10912580) were selected which can be tested by PCR-RFLP method. Genomic DNA was extracted from whole blood with the universal genomic DNA Extraction Kit VER.3.0 (TaKaRa, Japan). Genotyping was performed by polymerase chain reaction-restriction fragment length polymorphism (PCR-RFLP) method. The polymorphic region was amplified by PCR using a T-Gradient Thermoblock PCR System (Biometra, Germany) in a 25ul reaction solution containing 0.3ug genomic DNA (100ng/ul), 2.5ul 10× PCR buffer (Mg^2+^ plus) (TaKaRa, Japan), 2.0ul dNTPs mixture(TaKaRa, Japan), 0.25ul TaqDNA polymerase (5U/ul) (TaKaRa, Japan) and 0.1ul of each primer(10umol/L)(Invitrogen, China). Primers sequences of each SNP were listed in Table S3. The PCR products were digested with restriction enzymes (NEB, UK) according to the manufacturer’s instruction and analyzed by agarose gel electrophoresis. The accuracy of genotyping results were confirmed by direct sequencing in random samples.

### Statistical Analysis

Genotype frequencies of seven SNPs were tested for Hardy–Weinberg equilibrium (HWE), which was tested using the Chi-square test. Genotype frequencies were estimated by direct counting. The basic association test for a disease trait based on comparing allele frequencies between cases and controls was achieved by Plink 1.07 software (http://pngu.mgh.harvard.edu/~purcell/plink/). The asymptotic *P*-values are available. In addition to the allelic test of association, *P* values, odds ratios (ORs) and 95% confidence intervals (CIs) were calculated using logistic regression analysis adjusted for age, assuming an additive model (additive effect of having one additional copy of a allele, a was for the minor allele and A was for the major allele), dominant model (aa+Aa vs. AA) and recessive model (aa vs. Aa+AA) with Plink 1.07 software. Linkage disequilibrium, haplotype tests and multiple testing were analyzed with the Haploview 4.1 software (http://www.broad.mit.edu/mpg/haploview/), which constructs haplotypes based on the D’ values generating from our own data as the Solid Spine of LD method. Chi-square test was also performed to analyze the association between the SNPs and various clinical features of breast cancer with the Haploview 4.1 software. For accurate multiple testing correction, *P* values of the alleles and haplotypes were permutated 10,000 times using Haploview 4.1 software. All statistical tests were two-sided, and *P* values less than 0.05 were considered statistically significant.

## Supporting Information

Figure S1 **The pairwise D’ and Haplotype-block of the seven SNPs in OX40L gene.** Linkage disequilibrium (LD) strength was shown in the diamonds represented by D’ value, and bright red represent high-pairwise D’ value, which were generated by Haploview 4.1. Blocks were defined as the method of solid spine of LD according to the values of D’ generating from our own data.
(DOC)

Table S1 **Significant associations between OX40L SNPs and C-erbB2 status in cases.** SNP, single nucleotide polymorphism. C-erbB2, human epidermal growth factor receptor 2. ^a^ The number of cases with negative C-erbB2 was 286, and ^b^ the number of controls with positive C-erbB2 was 185. The *P* values were assessed under an additive model (additive effect of having one additional copy of a allele, a was for the minor allele and A was for the major allele), dominant model (aa+Aa vs. AA), and recessive model (aa vs. Aa+AA) using logistic regression adjusted for age with Plink 1.07. Significant values (*P*<0.05) are in bold.
(DOC)

Table S2 **Associations between haplotype of OX40L gene SNPs and C-erbB2 status in cases.** Blocks were constructed as the method of solid spine of LD according to the values of D’ generating from our own data. Haplotype data was analyzed using Haploview 4.1. Significant values (*P*<0.05) are in bold.
(DOC)

Table S3 **Information of primers and products.**
(DOC)
