# Supplementary material for: Association of OX40L Polymorphisms with Sporadic Breast Cancer in Northeast Chinese Han Population
Source: PLoS One. 2012 Aug 3;7(8):e41277. doi: 10.1371/journal.pone.0041277 (PMC3411723; doi:10.1371/journal.pone.0041277)
Supplement: Figure S1 — The pairwise D’ and Haplotype-block of the seven SNPs in OX40L gene. Linkage disequilibrium (LD) strength was shown in the diamonds represented by D’ value, and bright red represent high-pairwise D’ value, which were generated by Haploview 4.1. Blocks were defined as the method of solid spine of LD according to the values of D’ generating from our own data. (DOC) [file pone.0041277.s001.doc]

**Figure S1. The pairwise D’ and Haplotype-block of the seven SNPs in OX40L gene**


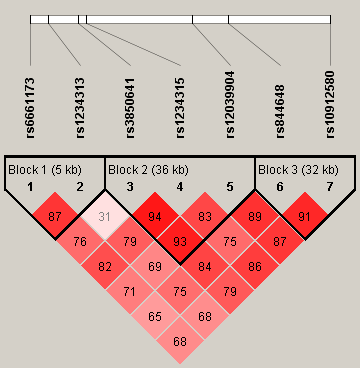


Linkage disequilibrium (LD) strength was shown in the diamonds represented by D’ value, and bright red represent high-pairwise D’ value, which were generated by Haploview 4.1. Blocks were defined as the method of solid spine of LD according to the values of D’ generating from our own data.
