# Supplementary material for: Association of OX40L Polymorphisms with Sporadic Breast Cancer in Northeast Chinese Han Population
Source: PLoS One. 2012 Aug 3;7(8):e41277. doi: 10.1371/journal.pone.0041277 (PMC3411723; doi:10.1371/journal.pone.0041277)
Supplement: Table S1 — Significant associations between OX40L SNPs and C-erbB2 status in cases. SNP, single nucleotide polymorphism. C-erbB2, human epidermal growth factor receptor 2. a The number of cases with negative C-erbB2 was 286, and b the number of controls with positive C-erbB2 was 185. The P values were assessed under an additive model (additive effect of having one additional copy of a allele, a was for the minor allele and A was for the major allele), dominant model (aa+Aa vs. AA), and recessive model (aa vs. Aa+AA) using logistic regression adjusted for age with Plink 1.07. Significant values (P<0.05) are in bold. (DOC) [file pone.0041277.s002.doc]

**Table S1. Significant associations between OX40L SNPs and C-erbB2 status in cases**

| SNP | Genotype | C-erbB2 status | | Allele | C-erbB2 status | | Additive *P* value | Allelic *P* value | Dominant *P* value | Recessive *P* value |
| --- | --- | --- | --- | --- | --- | --- | --- | --- | --- | --- |
|  |  | Negative^a^ | Positive^b^ |  | Negative | Positive |  |  |  |  |
| rs1234315 | TT | 89(31.12%) | 37(20.00%) | T | 307(53.67%) | 171(46.22%) |  |  |  |  |
|  | TC | 129(45.10%) | 97(52.43%) | C | 265(46.33%) | 199(53.78%) | **0.02896** | **0.02541** | **0.008199** | 0.3554 |
|  | CC | 68(23.78%) | 51(27.57%) |  |  |  |  |  |  |  |

SNP, single nucleotide polymorphism. C-erbB2, human epidermal growth factor receptor 2. ^a^ The number of cases with negative C-erbB2 was 286, and ^b^ the number of controls with positive C-erbB2 was 185. The *P* values were assessed under an additive model (additive effect of having one additional copy of a allele, a was for the minor allele and A was for the major allele), dominant model (aa+Aa vs. AA), and recessive model (aa vs. Aa+AA) using logistic regression adjusted for age with Plink 1.07. Significant values (*P* <0.05) are in bold.
