# Supplementary material for: Association of OX40L Polymorphisms with Sporadic Breast Cancer in Northeast Chinese Han Population
Source: PLoS One. 2012 Aug 3;7(8):e41277. doi: 10.1371/journal.pone.0041277 (PMC3411723; doi:10.1371/journal.pone.0041277)
Supplement: Table S2 — Associations between haplotype of OX40L gene SNPs and C-erbB2 status in cases. Blocks were constructed as the method of solid spine of LD according to the values of D’ generating from our own data. Haplotype data was analyzed using Haploview 4.1. Significant values (P<0.05) are in bold. (DOC) [file pone.0041277.s003.doc]

**Table S2. Associations between haplotype of OX40L gene SNPs and C-erbB2 status in cases**

| Block | Haplotype | Freq. | Positive,Negative Frequencies | Chi-square | *P* value |
| --- | --- | --- | --- | --- | --- |
| Block 1  (rs6661173  -rs1234313) | GA | 0.663 | 0.692, 0.645 | 2.199 | 0.1381 |
|  | GG | 0.271 | 0.244, 0.289 | 2.322 | 0.1275 |
|  | AG | 0.064 | 0.064, 0.064 | 0.00 | 0.9926 |
| Block 2  (rs3850641  -rs1234315  -rs12039904) | ACC | 0.472 | 0.517, 0.443 | 5.062 | **0.0245** |
|  | ATT | 0.245 | 0.231, 0.254 | 0.63 | 0.4273 |
|  | GTC | 0.132 | 0.120, 0.139 | 0.66 | 0.4166 |
|  | ATC | 0.125 | 0.106, 0.138 | 2.02 | 0.1553 |
|  | ACT | 0.015 | 0.013, 0.016 | 0.125 | 0.7232 |
| Block 3  (rs844648  -rs10912580) | GA | 0.513 | 0.540, 0.496 | 1.70 | 0.1923 |
|  | AG | 0.249 | 0.245, 0.251 | 0.049 | 0.8255 |
|  | AA | 0.213 | 0.204, 0.219 | 0.313 | 0.5761 |
|  | GG | 0.025 | 0.012, 0.034 | 4.388 | **0.0362** |

Blocks were constructed as the method of solid spine of LD according to the values of D’ generating from our own data. Haplotype data was analyzed using Haploview 4.1. Significant values (*P* <0.05) are in bold.
