# Supplementary material for: Association of OX40L Polymorphisms with Sporadic Breast Cancer in Northeast Chinese Han Population
Source: PLoS One. 2012 Aug 3;7(8):e41277. doi: 10.1371/journal.pone.0041277 (PMC3411723; doi:10.1371/journal.pone.0041277)
Supplement: Table S3 — Information of primers and products. (DOC) [file pone.0041277.s004.doc]

Table S3. Information of primers and products

| SNP | F(5’-3’) | R(5’-3’) | Annealing temperature(℃) | Restriction enzyme | Length of amplification product (bp) |
| --- | --- | --- | --- | --- | --- |
| rs6661173 | ACATAAAGCTTCCTCATTCC | CTTCCTCCAGGTTGATCTAC | 53 | BanI | 274 |
| rs1234313 | CTCCTACCATGTCTCAAAC | CTGTCTTCCACAGTCCTC | 55 | BsrDI | 304 |
| rs3850641 | GAACTGGTCTCTTTCCTATTTC | CCCACAGCAATCGTAAAG | 55.5 | HpyCH4III | 274 |
| rs1234315 | CACCAGGCTGGAAGTTTCAGGC | TAGCCAGACCTGGTGTTGCGTG | 55 | BsrI | 410 |
| rs12039904 | TGCTCATAGTTGCTTAATGC | AATAATCAGGCTGTGGAAAC | 55 | Mn1I | 229 |
| rs844648 | AGTTACACTATGTGGCGTTTA | CAGGCAGTTCCTCTTTGAT | 56 | AseI | 309 |
| rs10912580 | CAGGAGGATCATTTGAACC | CTTCGATGGAGACCATAAAC | 55.5 | AccI | 372 |
